# Successful abdominal wound closure for treatment of severe peritonitis using negative pressure wound therapy with continuous mesh fascial traction: a case report

**DOI:** 10.1186/s40792-018-0453-0

**Published:** 2018-05-09

**Authors:** Hideki Kogo, Jun Hagiwara, Shiei Kin, Eiji Uchida

**Affiliations:** 1 Department of Gastrointestinal and Hepato-Biliary-Pancreatic Surgery, Nippon Medical School, Graduate School of Medicine, 1-1-5 Sendagi, Bunkyo-ku, Tokyo, 113-8602 Japan; 2 Department Emergency and Critical Care Medicine, Nippon Medical School, Tokyo, Japan

**Keywords:** Negative pressure wound therapy, Mesh traction, Severe peritonitis

## Abstract

**Background:**

Surgery for severe peritonitis often entails difficult wound closure and may require open abdominal management due to gut edema and/or concern of abdominal compartment syndrome. Negative pressure wound therapy (NPWT) is known to have good outcomes for wound closure after surgery for severe peritonitis. NPWT with continuous mesh fascial traction may result in even better outcomes, especially for fascial closure.

**Case presentation:**

An 81-year-old man was hospitalized for abdominal pain. At admission, computed tomography (CT) demonstrated multiple liver metastases and a tumor perforating the sigmoid colon. Acute peritonitis due to perforated sigmoid colon cancer was diagnosed, and emergency peritonitis surgery and Hartmann’s operation were performed. However, at the end of the operation, the surgical abdominal wound could not be closed due to gut edema and concern of abdominal compartment syndrome. Thus, the abdominal wound was left open and NPWT was performed in the primary operation. In the second and subsequent operations, NPWT with mesh fascial traction was performed. The wound was ultimately closed in the fifth operation, which took place 9 days after the primary operation.

**Conclusions:**

Treatment of severe peritonitis requires that gastroenterological surgeons learn some form of open abdominal management. This case suggests that NPWT with fascial mesh traction is a suitable solution. Furthermore, it does not require any special materials, and surgeons will find it easy to perform. In sum, NPWT with fascial mesh traction may be the preferred method of open abdominal management over other techniques currently available.

## Background

Surgery for severe peritonitis often entails difficult wound closure and may require open abdominal management because of gut edema and/or concern of abdominal compartment syndrome. Abdominal wounds that are difficult to close after emergency surgery should be managed via an open abdominal technique; however, the longer the wound remains open, the more difficult it will be to close because, in median laparotomy incisions, the rectal muscle in the wound shrinks laterally with time. To increase the success rate of wound closure surgery and avoid subsequent complications, various open abdominal methods have been developed [1–4]. These methods have become indispensable in emergency abdominal surgery and continue to be improved upon. In surgery for severe peritonitis, negative pressure wound therapy (NPWT) is reported to result in good outcomes for wound closure [5]. Moreover, NPWT with continuous mesh fascial traction may have even better outcomes, especially for fascial closure [6–8]. Here, we present a case of severe peritonitis successfully treated by abdominal wound closure with NPWT and mesh fascial traction.

## Case presentation

**Fig. 1:**
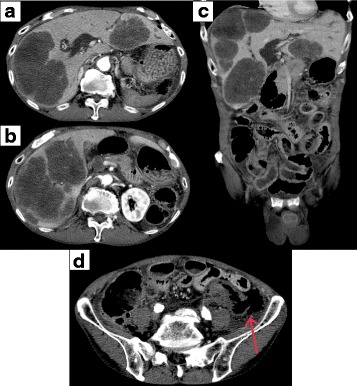
CT scan of the abdomen. **a**–**c** Multiple liver metastases were revealed. **d** Abdominal free air and perforating sigmoid colon cancer (arrow) were revealed

An 81-year-old man presented to our emergency department with abdominal pain that occurred after eating. At initial examination, the patient was alert but had difficulty standing due to significant abdominal distention and pain. His blood pressure was 118/68 mmHg with a regular pulse rate of 83 beats per minute. Laboratory test results included a white blood cell count of 3000/μL, hemoglobin concentration of 12.7 g/dL, platelet count of 241,000/μL, aspartate transaminase concentration of 177 IU/L, alanine transaminase concentration of 114 IU/L, lactic dehydrogenase concentration of 1105 IU/L, total bilirubin of 2.0 mg/dL, C-reactive protein concentration of 7.22 mg/dL, carcinoembryonic antigen (CEA) of 166.8 ng/mL, and CA 19–9 of 157.9 U/mL. A computed tomography (CT) scan of the abdomen revealed multiple liver tumors (Fig. 1a–c), including a tumor that perforated the sigmoid colon cancer with surrounding free air (Fig. 1d). Diagnosis was acute pan-peritonitis due to sigmoid colon cancer perforation with metastatic liver tumors and emergency surgery was performed.

### Operative findings

**Fig. 2:**
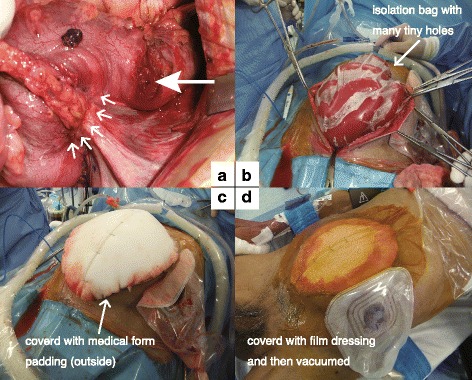
Findings at the primary emergency operation. **a** Primary tumor invasion of the retroperitoneum (arrow), with a perforated hole (large arrow). **b**–**d** Conventional NPWT was performed

**Fig. 3:**
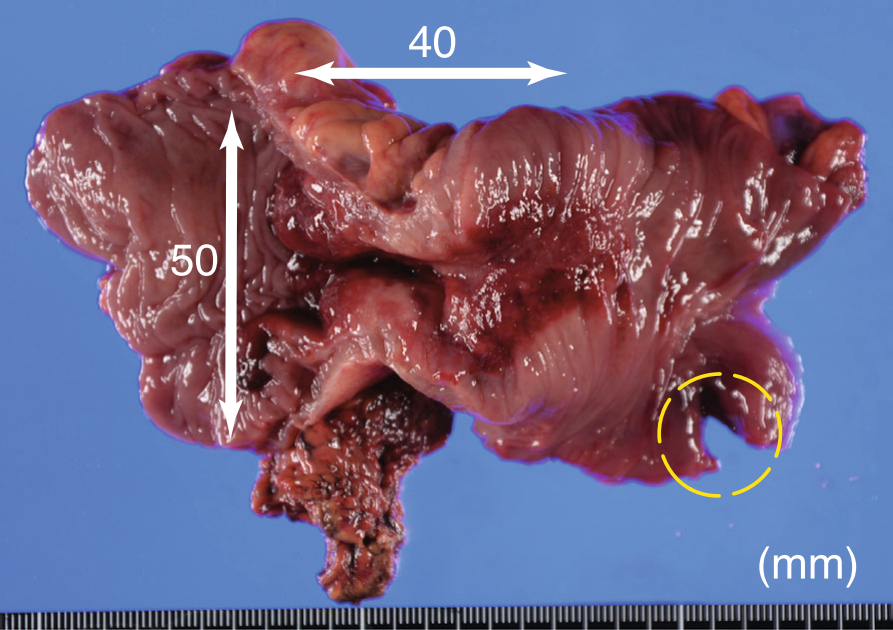
Colectomy specimen. Gross examination of the specimen revealed a large central tumor and a small perforated hole (dotted circle) adjacent to the tumor. Histopathology identified moderately differentiated adenocarcinoma of the colon (S, Type 2, 50 × 40 (mm), pT4b, int, INF-β, ly1, v2 (EMG), PN1b, pNx, pPM0 (15 mm), pDM0 (40 mm), RM1), categorized as stage IV, according to both the Japanese and TNM classifications

A median incision was made and dirty ascites, including fecal contamination, significant bowel edema, and multiple liver tumors, were noted. We located the perforating sigmoid colon tumor (Fig. 2a) and performed Hartmann’s operation. The sigmoid colon cancer perforation was noted in the resection specimen (Fig. 3). However, at the end of the operation, we were not able to close the wound due to gut edema and concern of abdominal compartment syndrome. Thus, the abdominal wound was left open and we performed NPWT (Fig. 2b–d). Following the operation, the patient was transferred to the surgical intensive care unit (ICU). Histopathological diagnosis was adenocarcinoma of the sigmoid colon.

### Postoperative course

**Fig. 4:**
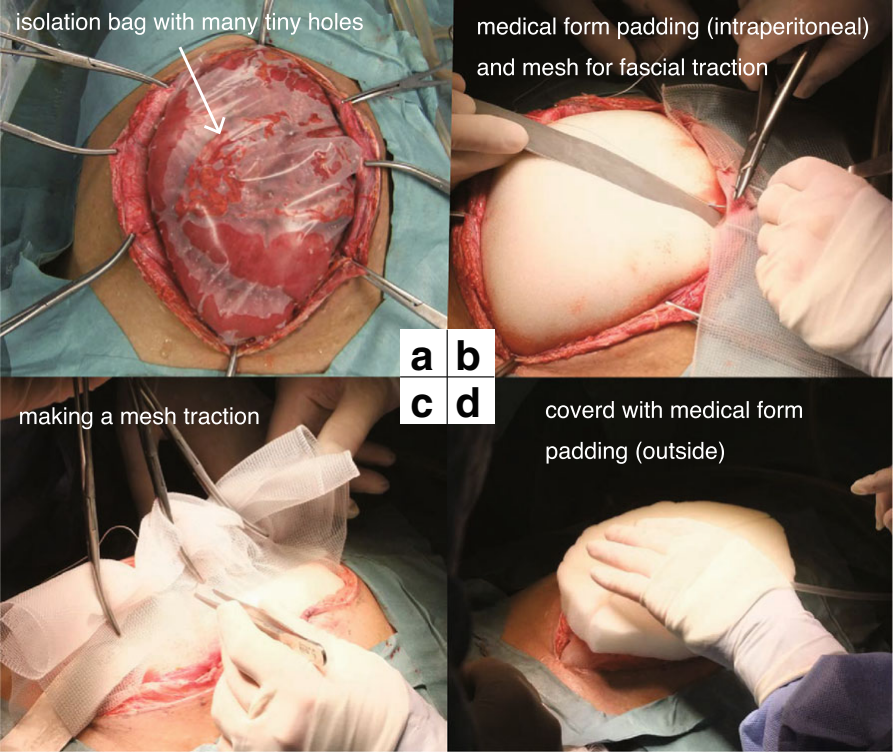
Findings at the second operation. The gut was edematous and the wound could not be closed. Thus, mesh traction was performed. **a** The gut edema did not improve at all. **b**, **c** Medical form padding (intraperitoneal) was introduced and mesh fascial traction was performed. **d** We covered the mesh traction with medical form padding (outside)

**Fig. 5:**
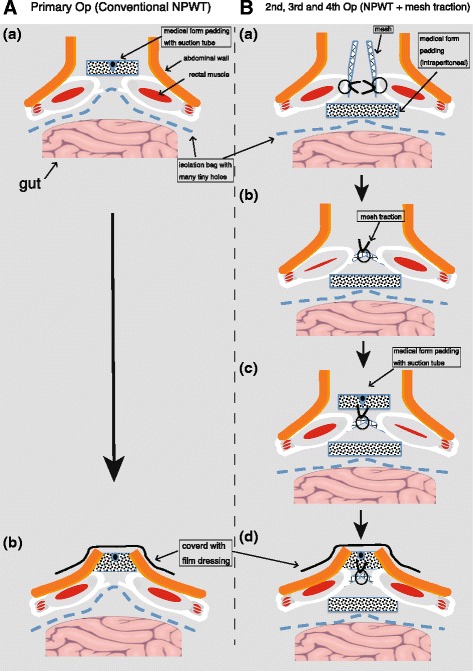
Operative schemes. **A** Conventional NPWT at the primary operation. **B** NPWT with mesh traction at the second through fourth operations

Two days after the primary emergency operation, a secondary operation was performed to determine whether the wound could now be closed. We concluded that it was still not possible to close the wound and performed continuous NPWT with mesh fascial traction (Figs. 4 and 5).

Five days after the primary operation, a third operation was performed. However, the wound still could not be closed. Thus, the abdominal space was irrigated, and three drains were inserted at the Douglas, Winslow, and left subphrenic spaces.

**Fig. 6:**
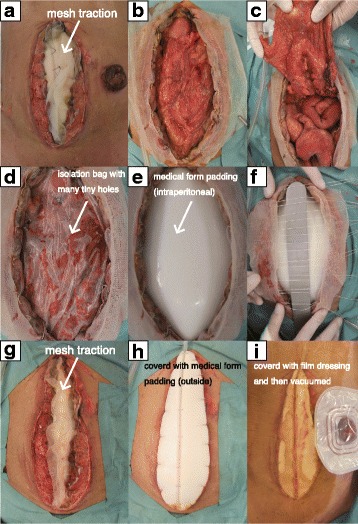
Findings at the fourth operation. Abdominal distension and gut edema had decreased. **a** Abdominal distension had decreased. **b**, **c** Gut edema also had decreased. **d**-**i** NPWT with mesh fascial traction was performed

A fourth operation was performed at 7 days and revealed improvement in the bowel edema. However, edema of the abdominal wall persisted. Thus, we irrigated the abdominal space and continued NPWT with mesh fascial traction (Fig. 6).

**Fig. 7:**
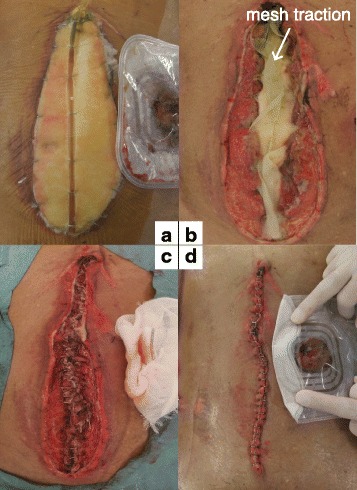
Findings at the fifth operation. **a** Prior to the operation. **b** The medical form padding was removed and the tension of the wound became free. **c**, **d** Fascial closure and skin closure could be performed

Nine days after the primary operation, a fifth operation was performed and revealed significant improvement in the bowel and abdominal wall edema. We were then able to close the wound without any complications (Fig. 7). The patient was weaned from mechanical ventilation and extubated 15 days after the primary operation. However, the growth of the multiple liver metastases could not be inhibited and jaundice did not improve. The patient died of the disease 18 days after the primary operation.

## Discussion

Common conditions that require open abdomen management include abdominal trauma, abdominal aortic surgery, and severe peritonitis [9, 10]. As peritonitis is often encountered in emergency surgery and the wounds sometimes cannot be closed in severe cases, surgeons must be familiar with some form of open abdominal management [11].

In this case, we performed NPWT with fascial mesh traction in a patient whose wound could not be closed following emergency peritonitis surgery. The wound was ultimately closed 9 days postoperatively without any complications, after a total of five operations. In many cases, the NPWT method requires film dressings to be changed every 2 to 3 days. Therefore, patients undergoing NPWT generally require frequent operative procedures. We also performed postoperative management in the ICU.

The effectiveness of mesh traction in this case is noteworthy. The reason for the difficulty of wound closure was intestinal edema as well as retraction of the rectal muscle laterally with time. Thus, it is important to maintain continuous muscle tension during treatment.

In the performed procedure, we excised the central portion of the mesh and sutured it back together to provide effective traction in each operation and subsequent narrowing of the defect (Figs. 4, 5B, and 6) [4]. This method does not require exchange of the mesh in each operation and makes it easy to adjust the tension of the mesh traction in a short time. Moreover, this method did not require any special materials and was easy to perform, even for surgeons who do not specialize in emergency surgery. In the fourth operation, the gut edema had significantly decreased. At that time, mesh fascial traction prevented the muscle from retracting laterally. Therefore, we were able to prepare for wound closure. Finally, in the fifth operation, we were able to close the wound.

NPWT is a highly effective method of wound closure with much better results than temporary closure [7, 12]. Furthermore, treatment outcomes (wound closure) have been shown to improve dramatically by adding mesh traction [6, 8, 13–16].

The current case demonstrated that NPWT plus mesh traction is the simplest and most effective treatment method currently available. Initial surgery is often finished with only NPWT. In cases where wound closure is judged to be difficult within a short time, adding mesh traction would increase the success rate. If the patient’s condition or intestinal edema is severe and long-term open abdominal management is likely to be needed, mesh fascial traction could be introduced in the initial surgery.

The current case involved a highly metastatic liver cancer, and the patient did not survive; however, if the cancer was not so advanced, the patient could have survived using this method.

## Conclusions

Gastroenterological surgeons must learn some form of open abdominal management for severe peritonitis. This case suggests that NPWT with mesh fascial traction is effective for wound closure in patients with severe peritonitis. Furthermore, this method did not require any special materials, was easy to perform, and may provide better open abdominal closure than any other technique currently available.

## Abbreviations

CT: Computed tomography
ICU: Intensive care unit
NPWT: Negative pressure wound therapy
POD: Postoperative day
